# Divergent seasonal responses of above- and below-ground to environmental factors in alpine grassland

**DOI:** 10.3389/fpls.2022.1091441

**Published:** 2023-02-06

**Authors:** Xiaojing Qin, Xiaojun Nie, Xiaodan Wang, Jiangtao Hong, Yan Yan

**Affiliations:** ^1^ School of Surveying and Land Information Engineering, Henan Polytechnic University, Jiaozuo, China; ^2^ Institute of Mountain Hazards and Environment, Chinese Academy of Sciences, Chengdu, China

**Keywords:** above-ground, below-ground, seasonal pattern, alpine steppe, synchronous

## Abstract

**Introduction:**

Under current global warming, the relationship between season changes of plants and environmental factors is focused on high-elevation and latitude regions. Due to the desynchronized growth of above- and below-ground and the buffering of soil, the driving factors in leaf and root show seasonal dynamics.

**Methods:**

We measured above- and below-ground intensity in the alpine steppe over the non-growing season (October-April) and growing season (May-September). Air temperature, precipitation, soil moisture, and soil temperature were used to analyze the correlation based on the growth rhythm.

**Results:**

Results showed that an earlier growth in spring and a delayed dormancy in autumn of root than leaf was observed. Our results strongly suggest that soil moisture plays a more important role in leaf unfolding while temperature is consistent with the withering of the shoots. Soil moisture comes from soil melt driving the spring phenology of roots, which derived from the storage of the subsoil layer in the last autumn.

**Discussion:**

Climate change will affect the strong seasonal patterns that characterized these precipitation-limited systems, especially in the spring and fall shoulder seasons. As seasonality changes in the alpine steppe, divergent responses of leaf and fine root would be explored.

## Introduction

Relationships between aboveground productivity or phenology and temperature and precipitation have been discussed in grassland ecosystems (Wu et al., 2013; Ren and Peichl, 2021; Shao et al., 2022), which indicates the changing trend of grassland under climate change (Sun et al., 2022). Evidence for this has been found from the local (Zhang et al., 2019) to the landscape scales (Chen et al., 2020), with methods ranging from single-shot observation (Chen et al., 2015) to remote sensing (Doussoulin-Guzmán et al., 2022). In high-latitude and high-altitude regions, annual aboveground cycles are constrained by a short growing season. Accordingly, the timing of root growth influences plant resource acquisition, which plays a key role in ecosystem function and biogeochemical cycles (Warren et al., 2014). An increasing number of studies have proven that the peak values of above- and belowground biomass did not occur at the same time (Blume-Werry et al., 2015), so the root and shoot growth in the nongrowing season and the driving factors should be understood.

In mountain areas, spring accounted for more than 50% of the annual production of roots, although peak root production may not be related to the high summer temperatures and the acquisition of nutrients (Garcia-Pausas et al., 2010). It has been proven that root length and root biomass peaking in summer relied on the lag effect of early-season accumulative precipitation (Cao et al., 2020). In view of the several factors determining the dynamics in seasonal belowground productivity, the thermal gradient along the profile in soil plays a key role (Korner and Paulsen, 2004). Due to the consistent alpine snow cover and the predominance of permafrost in alpine ecosystems, a seasonality delay of the soil physical environment was more obvious in the spring-summer and autumn-winter periods. Accordingly, the freeze-thaw cycles of the soil surface layer at the beginning and the end of the growing season could affect the root activity and the plant’s ability to grow. That is, the influence of the seasonal physical environment of air and soil on above- and belowground may be inconsistent.

Annual climatic factors are usually considered to be the seasonal patterns of above- and belowground productivity (Zheng et al., 2020; Wu et al., 2021). Regarding other factors, the effects of short-term microclimatic conditions on plants were especially relevant and sensitive, and they are critical to understanding carbon storage and turnover in grassland ecosystems (Cheng et al., 2022). For example, autumn precipitation and minimum temperature are primary climatic controls of alpine grassland autumn phenology (An et al., 2020). The gradually rising daily mean air temperature and accumulation of daily precipitation from late winter to early spring likely determine the spatiotemporal variations in green-up on the world’s roof (Chen et al., 2015). On the other hand, the microclimatic soil factors related to soil moisture and soil temperature should be taken into account for photosynthetically fixed carbon, as should leaf expansion and the duration of root production.

The following assumptions were examined in this study: We asked whether phenology and seasonal patterns were synchronized above and below ground in alpine grasslands. We also asked whether the influence of environmental factors, such as air temperature, precipitation, soil temperature, and soil moisture, on root and leaf growth dynamics differs in different seasons. To address these questions, we performed *in-situ* measurements of phenology and compared the growth curves of above and below ground during the nongrowing and growing seasons in northern Tibet and additionally measured air and soil temperature as well as soil moisture along the profile.

## Materials and methods

### Study area

The experiment was conducted at the Xainza Alpine Steppe and Wetland Ecosystem Observation Station, which is located in northern Tibet (N 30°57′, E 88°42′, 4675 m a.s.l). The site has a typical Plateau continental climate, which has a cold and semi-arid plateau monsoon climate. The average annual air temperature at the location is 0°C, and the average annual precipitation is 300 mm, which mainly falls during the summer growing season of June-August. Vegetation in our study area is dominated by *Stipa purpurea* and *Carex moorcroftii* and the accompanying species include *Artemisia nanschanica* and *Oxytropis glacialis*. More than 80% of the root biomass is located in the uppermost layer (0-30 cm). There is no absolute frost-free season and the frosty period lasted up to 280 days. In general, the freeze period started when the daily maximum air temperature at the soil surface was below 0°C, and finished when the daily minimum air temperature at the soil surface was above 0°C continuously for 48 h (Henry, 2007).

### Data collection and analysis

As an *in situ* method, minirhizotrons (M.G. Johnson, 2001) were used to directly view and study fine roots. Nine minirhizotron tubes (angled tubes with 45° in field settings) were installed in July 2017, to avoid soil disturbance and the artifacts in subsequent root data, and image collection started in March 2018 and ended in October 2018. A sampling interval of three days in the thaw period (March-April), one week in the growing season (May-July) and six days in the freezing period (October-November).

Root length was measured using the software Rootfly version 2.0.1 (Birchfield and Wells, Clemson University, Clemson, SC, USA) which is based on the community level. Root growth was calculated as the increase in root length (the appearance of new roots) between two sampling times for each tube. In this paper, we analyze the canopy photosynthesis by digital cameras which were fixed onto sticks 30-40 cm above the ground. Camera imageries were collected at an interval of one day to measure the canopy color metrics. We processed the image archives to extract regions of interest that encompassed all portions of the full canopy within the foreground in the time series. The increase of canopy within each sampling interval relative to the maximum percentage was used as above-ground production, whereas a loss of greenness was used as above-ground senescence. In view of the destructiveness of *in situ* sampling, shoot to root ratio was evaluated using the ratio between the above-ground cover and below-ground root length.

Soil temperature and moisture were recorded as hourly means with three sensors (Em 50, Decagon Devices, Pullman, WA, USA) at a soil depth of 10 cm, 20 cm, and 30 cm. Air mean, minimum, and maximum daily temperature and daily precipitation were calculated from the meteorological station.

Spearman’s rank correlation coefficients were used for correlations between abiotic factors (air temperature (mean, minimum, maximum), precipitation, soil temperature, and soil moisture (10 cm, 20 cm, 30 cm)) and plant pert (leaf coverage, root length, shoot to root ratio) in the different period intervals by the growth rate, following by SPSS Inc 16.0 completed. We used Surfer 13.0 and Sigmaplot 12.0 for modeling figures.

## Results

### Phenology and growing season length above and below

**Figure 1:**
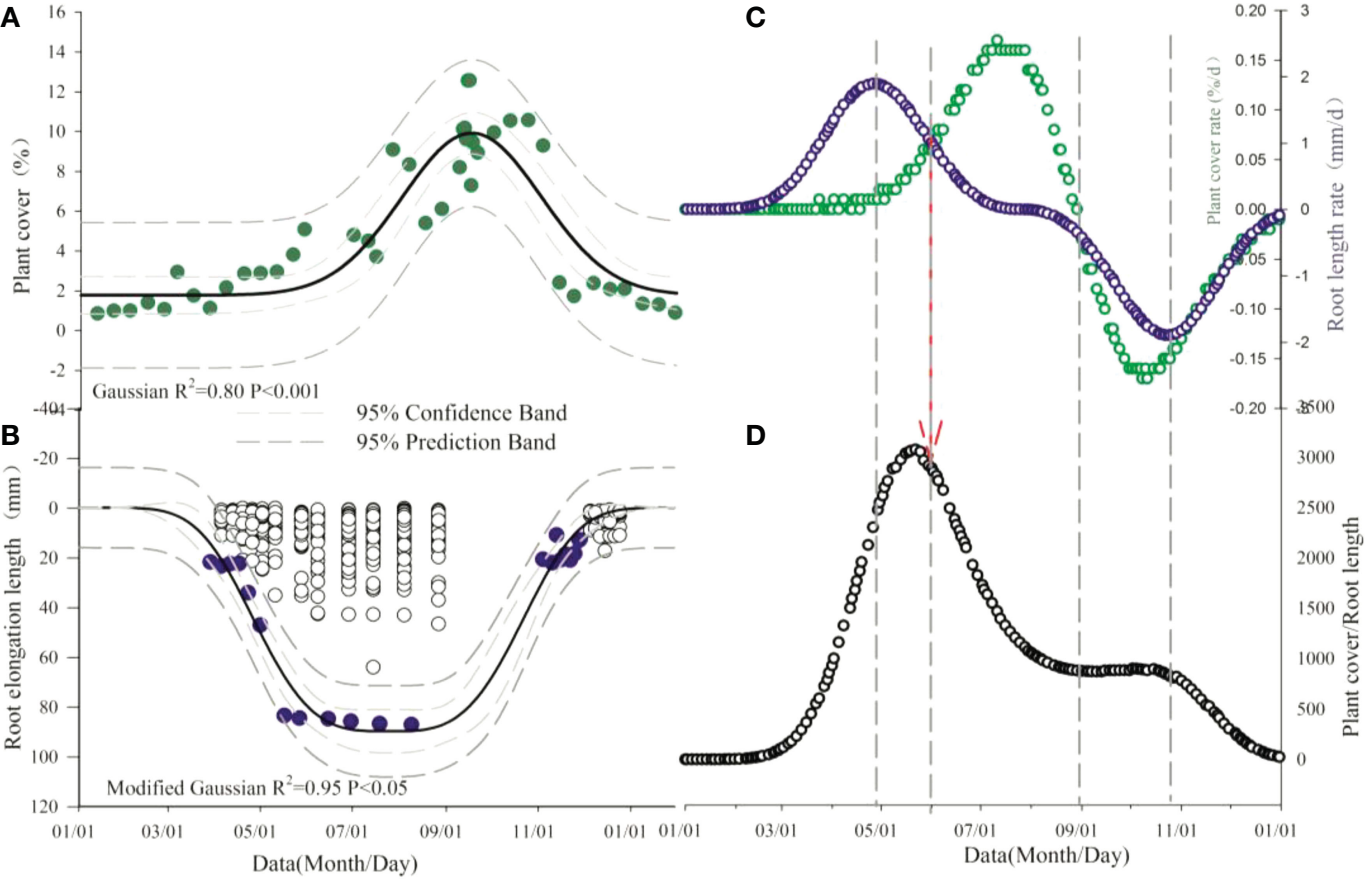
Seasonal dynamic of total vegetation cover **(A)**, root length **(B)** and growth rate of plant cover and root length **(C)** and shoot-to-root ratio **(D)**. Dashed arrows indicate the change point of relation between above- and below-ground.

Above- and below-ground production patterns were not synchronous (**Figure 1**). Shoot production peaked in the middle of August and declined soon afterwards, but below-ground production was more uniformly distributed over the growing season relative to above-ground production without a distinct peak in temperature grassland. The fit Gaussian curve in the alpine tundra inflected a longer and more evenly distributed production below ground than above ground. The allocation pattern of root and shoot (R/S) showed different seasonal changes, on account of the response to environmental factors.

This result (**Figure 1**) was confirmed the general growth length in alpine grassland, showing an earlier and faster start growing production below ground. After the end of above-ground production, the level of greenness immediately declined instead of remaining constant and thus coincided with the onset of above-ground senescence. Root elongation intensity, on the other hand, was still ongoing and consistently continued while there was a substantial loss of leaf senescence.

### Seasonality of the physical environment

**Figure 2:**
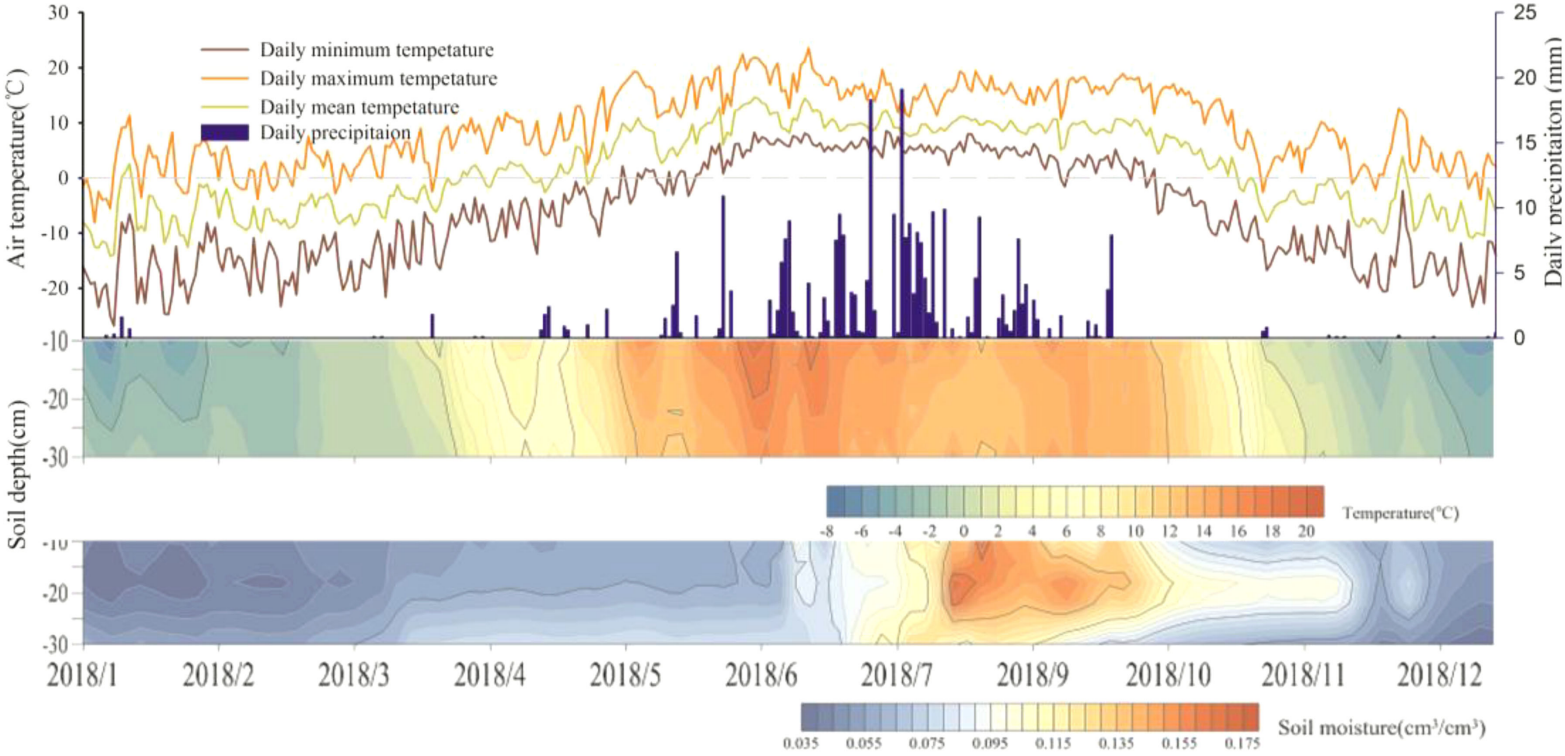
Diagram of the daily air temperature (mean, minimum and maximum), precipitation and soil temperature and soil moisture (10 cm, 20 cm and 30 cm).

Alpine grassland systems share a cold, short growing season, and the timing of onset and duration of the growing season is similar to the seasonality changes of the physical environment (**Figure 2**). In spring, the start of the growing season is cued by incoming air and soil temperatures, which also induce snow melt and soil thaw. The rainy season occurs after the increase in spring soil moisture by freeze-thaw-process. Air temperature was synchronized with topsoil temperature (0-30 cm) in the above-ground growing season, while average soil moisture showed coinciding peaks in August, which was still ongoing and continued to October. Soil moisture at 20 cm depth in the autumn measured center. Compared to air temperature, the time at which the soil temperature reached freezing point was earlier in spring and later in winter.

### Abiotic parameters influencing shoot and root growth

**Figure 3:**
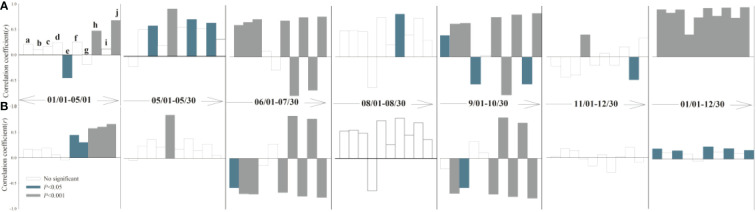
Partial correlation analysis between root length **(A)**, growth rate **(B)** and climatic factors (a: daily mean air temperature; b: daily minimum air temperature; c: daily maximum air temperature; d: daily precipitation; e: soil moisture -10 cm; f: soil temperature -10 cm; g: soil moisture -20 cm; h: soil temperature -20 cm; I: soil moisture -30 cm; j: soil temperature -30 cm). Correlation coefficient >0 denotes positive correlation, while correlation coefficient <0 denotes negative correlation.

**Figure 4:**
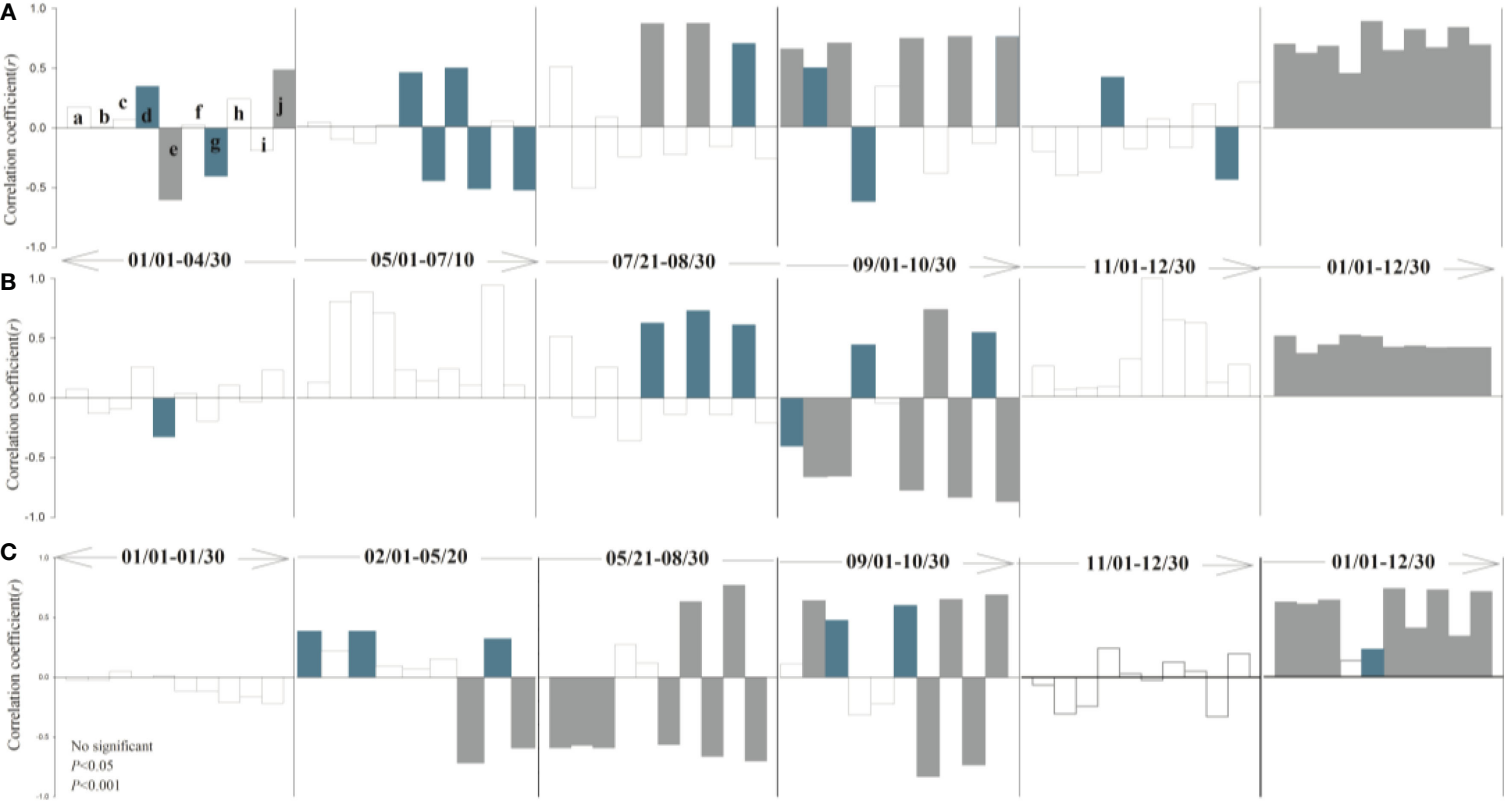
Partial correlation analysis between leaf canopy **(A)**, growth rate **(B)**, shoot-to-root **(C)** and climatic factors (a: daily mean air temperature; b: daily minimum air temperature; c: daily maximum air temperature; d: daily precipitation; e: soil moisture -10 cm; f: soil temperature -10 cm; g: soil moisture -20 cm; h: soil temperature -20 cm; I: soil moisture -30 cm; j: soil temperature -30 cm). Correlation coefficient >0 denotes positive correlation, while correlation coefficient <0 denotes negative correlation.

The response of leaf and root growth to air temperature, precipitation, soil temperature, and soil moisture varies seasonally among different periods (**Figure 3**, **4**). During the rapid root growth period (March-May), there was a positive significant correlation between root growth rates and soil temperature and soil moisture in soil depth of 20-30 cm, while the negative significant correlation was found between root elongation intensity and soil moisture in topsoil (10 cm). Spring soil moisture due to soil thaw provided a resource for root acquisition (Hong et al., 2017), while topsoil moisture may be affected by evaporation. Average air temperature and soil moisture played an important role in root elongation intensity in May, when the above-ground greenness increased. Yet, soil moisture (10-20 cm) drives the leaf growth, but the soil temperature offsets the vegetation cover expansion. The root growth rate was restrained by both air and soil temperature in June and July, while had no significant correlation with abiotic parameters in August. Similarly, phenology was synchronized root and leaf, according to the decreased autumn temperature. There were no significant correlations between plant and climate factors after the freezing point was reached. Overall, the temperature was the major factor that affected root growth rate in alpine grassland.

## Discussion

### Seasonal patterns of above- and belowground production

The growing season started earlier in the spring and lasted considerably longer in the autumn below ground than above ground in alpine grassland. Vegetation coverage showed a distinct peak in the middle of summer, while the root elongation intensity maximum was even more pronounced at the beginning of the season. Dynamics in seasonal belowground and aboveground production, such as relative productivity rates, were not synchronous, and peaks were found in other regions (Ernakovich et al., 2014; Blume-Werry et al., 2015; Cao et al., 2020). A loss of greenness with senescence correlated with a reduction in photosynthetic activity. Accordingly, root growth and turnover could be explained by the functional plasticity in response to changing environmental conditions (Warren et al., 2014; Hong et al., 2017). Higher specific root length in the nongrowing season (October-April) than in the growing season (May-September) has been proven (Cao et al., 2020). The phenomenon that fine roots take up nutrients and grow in early spring also corresponds to our own observations. Similarly, root elongation intensity during late summer and early autumn does not return to early summer rates in forest ecosystems (J. D. Joslin, 2001). One possible explanation for this is that carbon allocation to sugars and starch of fine roots reflects their role in structural support and storage in late summer (Keel et al., 2012).

Grassland primary production would be underestimated if above- and below-ground part at the same time, due to the pattern and timing of root production and high turnover rate. We found that grassland and tundra were less coupled in habitats with leaf and root production than forests were (Steinaker and Wilson, 2008). Root observations in our study were mainly of fine roots (<2 mm), which strongly influences both the seasonal potential for soil resources and water acquisition and the plant fluxes of root carbon into soil pools (Mccormack et al., 2014). Conceptually, the response of fine roots in inherent relative growth rate to short-term changes was sensitive to root traits and morphology (Iversen et al., 2014). Under the current climatic change, fine root responses among different ecosystem communities and whether these communities become more uniform should be understood in the future (Chen et al., 2018).

#### Abiotic parameters influencing shoot and root growth

Grassland primary production has been reported to be related to climate parameters such as temperature, but in our study, there was a significant positive correlation between soil moisture, not air temperature or precipitation, and canopy greenness from May to August. Soil temperature even offsets the leaf growth rate at the beginning of the season. Herbaceous plant leaf traits are affected by the key controls of soil in semiarid regions (Zhang et al., 2022). In contrast, the temperature of grassland roots and leaves increased significantly with soil temperature (Steinaker and Wilson, 2008). Therefore, warmer winters were not found to induce a later green-up. This may be due to the different effects of temperature on leaves, roots, and soil in alpine ecosystems, all of which are affected by the different key environmental factors. Continued increases in temperature enhanced precipitation transpiration and reduced soil moisture from May to July. For the alpine steppe, there was less soil moisture supply than in the alpine meadow, and the alpine steppe was more sensitive to water but not to temperature (Xu et al., 2016).

The cumulative precipitation of the early season had a lag effect on root traits, so it was not necessary for plants to increase their root length to capture more water as the growing season precipitation increased (Zhang et al., 2019). Soil water availability in the surface soil layer is generally more affected by evaporation than that in the deeper soil. Soil moisture in the subsurface layer (10-30 cm) was more closely correlated with root length. The layer effects were obvious at high altitudes or latitudes in grassland (Cao et al., 2020). Therefore, root biomass which is located in the upper soil was also significantly correlated with cumulative precipitation. A previous study showed that the relative productivity rate, renewal time, and contribution to total belowground biomass were higher at the subsurface (5-15 cm) than at the surface layer (5 cm), which experiences freeze−thaw events in spring (Garcia-Pausas et al., 2010).

#### Microclimate conditions due to freeze−thaw cycles

On the one hand, increases in the frequency and high temperatures of late-winter warming events are likely to trigger early episodes of soil thaw, possibly reducing the capacity of plants to take up nutrients in early spring (Chen et al., 2015). On the other hand, our results presented the storage effect of soil in late autumn, providing water to roots in early spring, and the effect of soil-vegetation-atmosphere has been proven using observations, numerical experiments, and models (Yang and Wang, 2019). The degradation of permafrost was presented as manifested by ground surface thaw settlement (Sun et al., 2022). The manipulation of freeze−thaw cycles enhanced the release of nutrients, enabling roots to acquire resources to maintain life (Henry, 2007; Song et al., 2017).

In alpine ecosystems, decreased water availability from earlier snow melt and warmer summer temperatures lead to earlier senescence, indicating divergent responses to climate change, particularly in the spring and fall shoulder seasons (Ernakovich et al., 2014). This agreement with our results may be due to the important role of the freeze−thaw process. Climate change alters the seasonal patterns of temperature and precipitation, including the soil microclimate conditions. The ways in which species and communities respond to the changes in the seasonal timing and synchrony of events, or seasonality (Warren et al., 2014), are central to understanding and predicting responses in community structure and composition to global changes.

## Conclusion

This study provides a basic understanding of the seasonal dynamics of root length intensity and leaf canopy in the alpine steppe, and it also proposes the impact of air temperature, precipitation, soil temperature, and soil moisture on above- and belowground part, which can help explain the divergent in plant seasonal response to the climate. It is worth noting that in this study, soil moisture was a major driver of leaf unfolding, while the temperature was related to greenness loss in autumn. Root length intensity, while relies on the lag effect of subsoil moisture, comes from soil melt, and its peak value was earlier than that of leaves in that peak month. If more experiments can be performed at the community level, the response that was not synchronized with seasonal dynamics above and below ground to soil moisture and temperature can be deeply understood under current global warming.

## Data availability statement

The original contributions presented in the study are included in the article/supplementary material. Further inquiries can be directed to the corresponding author.

## Author contributions

Conceptualization, XW and XQ; Methodology, software, investigation, data curation, XQ and YY; Writing—original draft preparation, XQ; writing—review and editing, JH and XN; funding acquisition, XW. All authors have read and agreed to the published version of the manuscript.
